# Establishing a framework for privacy-preserving record linkage among electronic health record and administrative claims databases within PCORnet^®^, the National Patient-Centered Clinical Research Network

**DOI:** 10.1186/s13104-022-06243-5

**Published:** 2022-10-31

**Authors:** Daniel Kiernan, Thomas Carton, Sengwee Toh, Jasmin Phua, Maryan Zirkle, Darcy Louzao, Kevin Haynes, Mark Weiner, Francisco Angulo, Charles Bailey, Jiang Bian, Daniel Fort, Shaun Grannis, Ashok Kumar Krishnamurthy, Vinit Nair, Pedro Rivera, Jonathan Silverstein, Keith Marsolo

**Affiliations:** 1 Department of Population Medicine, Harvard Medical School and Harvard Pilgrim Health Care Institute, Boston, MA 02215 USA; 2 Louisiana Public Health Institute, New Orleans, LA 70112 USA; 3 Datavant, San Francisco, CA 94104 USA; 4 Cohen Veterans Bioscience, New York, NY 10018 USA; 5 Duke Clinical Research Institute, Duke University School of Medicine, Durham, NC 27710 USA; 6 Scientific Affairs, HealthCore, Inc., Wilmington, DE 19801 USA; 7 Department of Medicine, Weill Cornell Medicine, New York, NY 10021 USA; 8 Department of Medicine, Cook County Health and Hospital System, Chicago, IL 60612 USA; 9 Applied Clinical Research Center, Department of Pediatrics, Children’s Hospital of Philadelphia, Philadelphia, PA 19104 USA; 10 College of Medicine, University of Florida, Gainesville, FL 32610 USA; 11 Center for Outcomes and Health Services Research, Ochsner Health, New Orleans, LA 70121 USA; 12 Regenstrief Institute, Indiana University, Indianapolis, IN 46202 USA; 13 University of North Carolina, Chapel Hill, NC 27599 USA; 14 Sharon, MA 02067 USA; 15 OCHIN, Inc., Portland, OR 97201 USA; 16 Department of Biomedical Informatics, University of Pittsburgh, Pittsburgh, PA 15206 USA; 17 Department of Population Health Sciences, Duke Clinical Research Institute, Duke University School of Medicine, Durham, NC 27710 USA

**Keywords:** Medical record linkage, Multicenter studies, Patient data privacy

## Abstract

**Objective:**

The aim of this study was to determine whether a secure, privacy-preserving record linkage (PPRL) methodology can be implemented in a scalable manner for use in a large national clinical research network.

**Results:**

We established the governance and technical capacity to support the use of PPRL across the National Patient-Centered Clinical Research Network (PCORnet^®^). As a pilot, four sites used the Datavant software to transform patient personally identifiable information (PII) into de-identified tokens. We queried the sites for patients with a clinical encounter in 2018 or 2019 and matched their tokens to determine whether overlap existed. We described patient overlap among the sites and generated a “deduplicated” table of patient demographic characteristics. Overlapping patients were found in 3 of the 6 site-pairs. Following deduplication, the total patient count was 3,108,515 (0.11% reduction), with the largest reduction in count for patients with an “Other/Missing” value for Sex; from 198 to 163 (17.6% reduction). The PPRL solution successfully links patients across data sources using distributed queries without directly accessing patient PII. The overlap queries and analysis performed in this pilot is being replicated across the full network to provide additional insight into patient linkages among a distributed research network.

**Supplementary Information:**

The online version contains supplementary material available at 10.1186/s13104-022-06243-5.

## Introduction

PCORnet^®^, the National Patient-Centered Clinical Research Network [1, 2], is a network-of-networks developed with funding from the Patient-Centered Outcomes Research Institute^®^ (PCORI^®^). At the time of this work, it was comprised of Clinical Research Networks (CRNs) [3–11], with health systems or academic medical centers as members (Network Partners), Health Plan Research Networks (HPRNs)^1^ and a Coordinating Center. PCORnet leverages electronic health records (EHRs) and administrative claims data to conduct multi-center comparative effectiveness studies.

As patients in the United States may receive care at different unaffiliated health systems, individual sites do not always have complete capture of the necessary variables or outcomes of interest for many types of studies. One way to bridge this gap is to link overlapping records across Network Partners in a privacy-preserving manner. Individual CRNs participating in PCORnet had previously developed solutions that demonstrated the feasibility of conducting privacy-preserving record linkage (PPRL) [12–15]. Relying on individual, network-specific linkage is inefficient and unsustainable at scale, however, as cross-network analyses would require multiple local governance and technical solutions to be implemented each time a different solution was utilized.

To address this, PCORnet assembled a multi-disciplinary team to advise on how to establish a standardized and scalable PPRL infrastructure for the entire network. This group recommended identifying an existing solution to meet the Network’s needs. Through a competitive solicitation process, Datavant was selected to provide a PPRL solution for PCORnet.

We describe the PPRL solution, governance considerations and preliminary results from an overlap analysis that determines the unique, de-duplicated count of patients across the network and the generation of a summary-level table of patient characteristics describing the PCORnet population.

## Main text

### Methods

#### Token generation

The Datavant solution enables PPRL through the use of de-identified tokens that consist of keyed, salted hashes meeting the definition of de-identification through the Expert Determination Standard of the Health Insurance Portability and Accountability Act’s (HIPAA) Privacy Rule [16]. (In the United States, HIPAA defines two approaches by which a dataset can be considered de-identified—Safe Harbor, which requires the removal of specific identifiers, or Expert Determination, which offers more flexibility but requires an analysis to demonstrate the results are statistically de-identified.) Tokens are based on different permutations of personally identifiable information (PII). The PII is passed through a one-way FIPS 140-2 secure hashing function with the addition of a Datavant Master Salt, which irreversibly destroys the underlying PII (i.e., cannot regenerate from the hash values). The salted hashes are then encrypted using a site-specific encryption key to generate a set of site-specific tokens, ensuring each site’s tokens remain unique and safe from a security breach at another site. Finally, these site-specific tokens are transformed into transit tokens, where a second encryption key is assigned for interoperability with a specific token recipient (e.g., the Coordinating Center). Within PCORnet, these tokens are stored in the HASH_TOKEN table of each site’s PCORnet Common Data Model (CDM). [17]

#### Governance

PCORnet’s approach to governance was informed by PPRL best practices and experience on prior initiatives [13, 18–27]. Each PCORnet site maintains a local IRB protocol governing their instance of the PCORnet CDM. The local protocol describes the source system(s), the process for responding to Network queries, and other local requirements. To allow for hash tokens to be included in the CDM, a draft IRB amendment was shared with the Network describing the process for generating the tokens and generic workflow for linkage queries. To address concerns that linkage activities would occur without additional oversight, PCORnet Network Partners also decided that all linkage queries would be governed by their own IRB protocols. As a result, a second protocol was written to govern the overlap activities described here.

The transfer of data between the sites and Coordinating Center was covered by the PCORnet Master Data Sharing Agreement. This agreement covers the transfer of aggregate data, de-identified datasets, limited datasets containing patient-level records, and datasets that include PHI other than in a limited dataset. No patient data were transferred to Datavant as part of this project.

#### Matching strategy

To conduct the linkage, a query was distributed that extracted records from the HASH_TOKEN table within each site’s CDM. A unique patient reference ID was created for each patient record based on the site’s CDM network identifier (DataMart ID or DMID) and the patient pseudo-identifier (Patient ID or PATID) (Additional file 1: Table S1). Sites returned their HASH_TOKEN extract via a secure file transfer method and were downloaded by approved personnel in the Duke Clinical Research Institute (DCRI) portion of the Coordinating Center for PCORnet. The tokens were processed, and the Datavant Match software was executed to determine overlap.

We selected a matching strategy that declared records to be a match if the majority of available tokens in both records were the same. The Match software output a table consisting of the Datavant MatchID (e.g., master patient id), the PCORnet reference ID (DMID_PATID), and the encrypted tokens (Additional file 1: Table S2). A file containing only the MatchID and PCORnet reference ID (“Match Index”) was sent to the Harvard Pilgrim Health Care Institute (HPHCI) portion of the Coordinating Center for PCORnet to allow them to perform the necessary analyses. HPHCI did not receive any of the encrypted tokens.

#### Overlap analysis

Using the Match Index, we calculated the percentage of patients who appeared in 1, 2, 3, or ≥ 4 sites. We also calculated the percentage of patients reported as a match within each site’s records to assess potential duplicate patients or mismatches.

We calculated the percentage of patients in common for each pairwise comparison between sites. The percentage was calculated twice for each site-pair using the count of overlapping patients as the numerator both times and the count of patients from each site respectively as the denominator. We report a summary of the percentages including the minimum, maximum, average, and median.

#### Patient demographic characteristics table

**Fig. 1:**
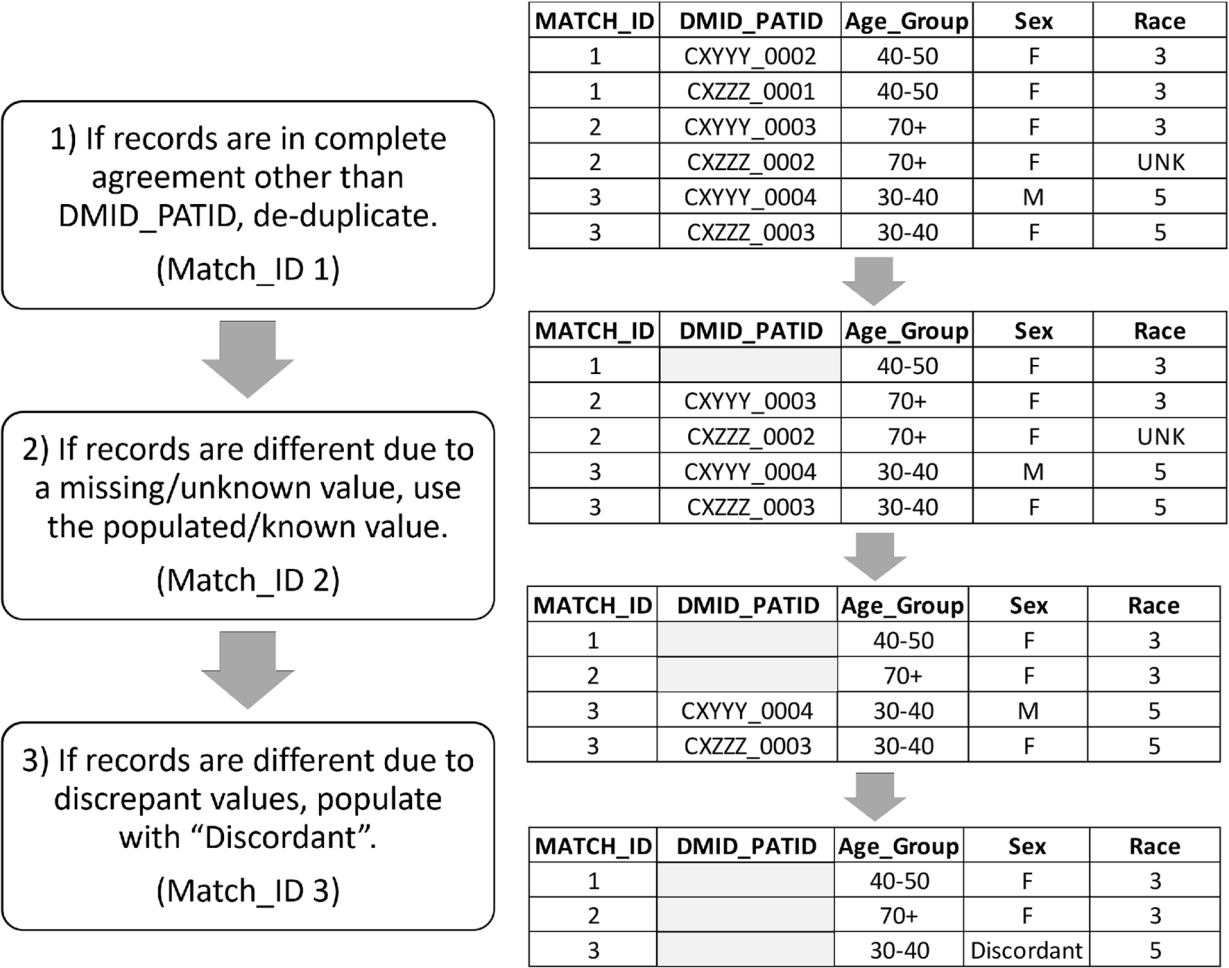
This process flow represents the records adjudication steps performed to de-duplicate records with patients with a common “Match_ID”. “MATCH_ID” is generated by the Datavant software and each represents a unique patient. “DMID_PATID” is the internal DM pseudoidentifier (but does not contain patient identifiers). “Age_Group” is the age category assigned to the patient based on patient age. “Sex” includes example Male and Female values from the PCORnet CDM. “Race” includes example values from the PCORnet CDM, where 01 = American Indian or Alaska Native, 03 = Black or African American, and 05 = White

We created a distributed query to retrieve basic demographic information for patients with any clinical encounter in calendar year 2018 or 2019. Sites returned these de-identified demographic characteristics via a second secure file transfer method and the files were downloaded by approved personnel in the HPHCI portion of the Coordinating Center. HPHCI used these data, along with the Match Index, to perform an overlap analysis and create the linked summary table. We generated a table of patient characteristics using the full dataset (i.e., with potential duplicates) and again after consolidating matched records. We applied a series of adjudication steps to consolidate matched records while retaining distinct information among records with discrepant values (Fig. 1).

Additional information on the Token Generation and Governance can be found in the Additional file 1.

## Results

### Token generation

The PII used for PCORnet Network Partner token generation were selected based on their overall availability across PCORnet member sites and their utility in determining overlap. Tokens generated using full address information were also considered, but not included due to limitations in the ability to standardize and normalize address data. We generated six tokens using a variety of PII combinations, including two tokens generated using social security number (SSN) (Table 1). PII elements such as first and last name, gender and date of birth are present for almost 100% of patients. Availability of SSN was more variable, with a range of 0% to 99% across partners (median 66%), but it was still included due to the sensitivity of SSN in determining matches when accurate values are available.

**Table 1:** Personally identifiable information used to generate the hash tokens

Token	Personally identifiable information used
Token 1	Last name + First initial of first name + Gender + Date of birth
Token 2	Last name (soundex) + First name (soundex) + Gender + Date of birth
Token 3	Last name + First name + Date of birth + 3-digit zip code
Token 4	Last name + First name + Gender + Date of birth
Token 5	Social security number + Gender + Date of birth
Token 6	Social security number + First name

Soundex is an algorithm that represents names as they sound in English rather than as they are spelled, which can allow for matches even with slight spelling variations (e.g., Jon and John). In the United States, Social Security is a federal social insurance program, and given that SSNs are often assigned at birth, these numbers are often used as a proxy for a national identification number.

In a methodological assessment conducted by Centers for Disease Control and Prevention (CDC) National Center for Health Statistics that compared “traditional” identifiable linkages between the CDC National Death Index and the CDC National Hospital Care Survey with PPRL using Datavant, the kappa statistic demonstrated near perfect concordance (kappa of 0.81–1.00) and similar match rates. This assessment used 5 of the tokens within the PCORnet set, and an additional token providing further First Name granularity. [28]

### Overlap analysis

We piloted our approach in an initial analysis of four sites, two each from two CRNs (two of these sites also piloted the matching process using synthetic data). Sites were selected based on regulatory and technical readiness and the expectation of a non-zero overlap. A total of 3,111,792 patients were found to have had any encounter in 2018 or 2019. Following the deduplication process, the total unique patient count was 3,108,515 (0.11% reduction). Nearly all patients appear within only one of the four sites (99.9%), with 0.1% of patients appearing in 2 sites. A nominal number of patients were matched to records within the same site, but these “duplicate” records were less than 0.0% of patient count for each of the four sites.

We also calculated the percent of patients in common for each pairwise comparison of participating sites and for sites grouped into their respective CRNs. Of the six site-pairs, three had overlapping patients. The maximum overlap among these was 0.2% of a site’s overall patient count and the minimum, average, and median values were all 0.0%. Site patient counts ranged from 417,251 to 1,094,272 patients.

### Patient demographic characteristics table

Table 2 shows the patient characteristics before and after de-duplication. The demographic distribution of patients remains consistent following de-duplication, with the largest reduction in count for patients with an “Other/Missing” value for Sex; from 198 to 163 (17.6% reduction).

**Table 2:** Aggregated and De-duplicated patient records, results from 4 PCORnet^®^ sites

	Aggregated		De-duplicated	
	N	%	N	%
Number of Patients	3,111,792		3,108,515	
Demographics				
By Age (N, %)				
0–11	940,050	30.2%	938,890	30.2%
12–19	554,156	17.8%	553,601	17.8%
20–34	434,342	14.0%	433,654	14.0%
35–49	367,632	11.8%	367,308	11.8%
50–64	395,326	12.7%	395,045	12.7%
65–74	248,587	8.0%	248,423	8.0%
≥ 75	171,699	5.5%	171,592	5.5%
By Sex (N, %)				
Female	1,672,689	53.8%	1,670,951	53.8%
Male	1,438,905	46.2%	1,437,399	46.2%
Other/Missing	198	0.0%	163	0.0%
Discordant			0	0.0%
By Race (N, %)				
American Indian or Alaska Native	6,576	0.2%	6,570	0.2%
Asian	99,478	3.2%	99,384	3.2%
Black or African American	589,916	19.0%	589,548	19.0%
Native Hawaiian or Other Pacific Islander	2,582	0.1%	2,580	0.1%
White	1,933,687	62.1%	1,931,937	62.1%
Other/Missing	479,553	15.4%	478,433	15.4%
Discordant			63	0.0%

## Discussion

The initial queries, linkage, and overlap analysis demonstrate the success of the PPRL solution in linking patients across varied data sources without directly accessing patient PII. The linkage solution allows studies to obtain de-duplicated patient counts and comprehensive capture of health records for an individual within a study population.

### Governance

Datavant has received an Expert Determination that the tokens generated by their software and used by PCORnet constitute a de-identified dataset under HIPAA. This determination allows for the querying and return of token-only datasets, enabling rapid linkage and quantification of overlap populations among potential data sources without the need for extensive data use agreements. Projects that wish to create a single dataset that combines tokens with additional variables would need to go through another Expert Determination process to claim that it is also de-identified (datasets with a sufficiently long list of variables or the inclusion of rare conditions/events may not be amenable to this process, however). To avoid the time and cost of this additional step, the PCORnet Coordinating Center separated the linkage tasks between the DCRI and HPHCI teams, ensuring that each group was working with a de-identified dataset. Neither Coordinating Center team had access to the full linked dataset.

## Limitations

Given the sensitivity of certain PII (e.g., SSN), it was not expected that all participating sites would submit a full set of tokens. Although the Datavant Match software allows for matches to be made using a variety of algorithms and token weighting, the sensitivity or specificity of matches may be reduced based on the tokens available. We chose a conservative approach in assigning matches using a majority of available tokens with equal weight. A more flexible matching logic that required matches on fewer tokens could potentially increase the number of matched patients with a slightly reduced confidence in the match. Relying solely on Token 2, for instance, would reduce the chance that spelling errors would cause a mismatch at the risk of also declaring different patients with similar names to be a match. There is no single best strategy for matching, however, and we believe it should be tailored to the research question and underlying patient population(s).

The sites included in the pilot were selected mostly based on their readiness to respond, instead of an expectation of a high percentage of overlapping patients (a non-zero overlap was expected, however, and the numbers did meet expectations). Inclusion of data from the remaining sites will provide additional insight into the characteristics of sites with a high degree of overlap (e.g., geographic proximity) and the overall volume of patient linkages across the network. Expansion to the full network will also incorporate data from administrative claims, allowing for clearer understanding of the types of data available in each source and the potential information gain that can be achieved via PPRL.

## Supplementary Information

**Additional file 1:** **Figure S1. **Overview of the tokenization and linkage flow within PCORnet. The following steps are used to complete the linkage tasks: 1) **Run Datavant tokenize.** Each site runs **Datavant** application in tokenize mode on-premises to generate tokens in their own site-specific token encryption scheme. Every site’s tokens are unique. A security breach at one site would not propagate across other sites in the ecosystem. No linking can happen without a site’s permission. 2) **Run Datavant transform-tokens.** Each site runs **Datavant** application in transform-tokens **to** mode to prepare tokens for sending to the Coordinating Center (CC); tokens are uniquely encrypted in transit. 3) **Run Datavant transform-tokens.** CC runs **Datavant** application in transform-tokens **from** to transform tokens into a common CC encryption scheme. Thus, tokens can only be linked at the CC. 4) **CC Performs Overlaps. **CC runs **Datavant** Match to determine overlap among records. **Figure S2.** Overall data flow for the linkage query. Partners generate de-identified tokens from PII held within their source systems (a). The Token Team of the Coordinating Center distributes a SAS query to extract tokens from the HASH_TOKEN table of the CDM (b). Partners execute the query against their CDM and upload the results to a Secure File Transfer location. These tokens are processed by the Datavant software solution (c) and then a Match Index is generated by executing the Datavant Match software (d). A version of this Index is passed to the Query Team of the Coordinating Center that does not include the underlying tokens (e). The Query Team distributes a SAS query to extract Demographic data. Partners execute it against the CDM and the results are returned to a second Secure File Transfer location (f). The results are pulled down by the Query Team and then combined with the token-less Match Index to complete the overlap analysis and to generate the summary demographic table (g). For this study, DCRI acted as the Token Team of the Coordinating Center and HPHCI as the Query Team. The number of participating Partners in this initial pilot was 4. **Table S1.** Illustration of the content of the HASH_TOKEN extract. There is one row per patient. DMID_PATID corresponds to the ID of the contributing DataMart and PATID corresponds to a pseudoidentifier used to link across all information belonging to a patient within the DataMart’s CDM. **Table S2.** Example of matching output. MATCH_ID is used to denote patients that match across DMs. Each MATCH_ID corresponds to a unique patient. DMID_PATID is the internal DM identifier (but does not contain patient identifiers). The TOKEN columns are populated with encrypted hash tokens. MATCH_ID and DMID_PATID are needed to perform the overlap analysis and generate the de-duplicated patient demographic characteristics table.

## Data Availability

A dataset was created for this work, but it is not available due to restrictions imposed by the original data holders. The governance surrounding the project required that the patient-level data be destroyed after analysis, but the underlying queries and source data do exist to allow it to be recreated.

## Abbreviations

PPRL: Privacy-Preserving Record Linkage
PCORI^®^: Patient-Centered Outcomes Research Institute®
PCORnet^®^: National Patient-Centered Clinical Research Network
CRN: Clinical Research Network
HPRN: Health Plan Research Network
PII: Personally-identifiable information
EHR: Electronic health record
HIPAA: Health Insurance Portability and Accountability Act
CDM: Common Data Model
DCRI: Duke Clinical Research Institute
HPHCI: Harvard Pilgrim Healthcare Institute
SSN: Social Security Number
CDC: Centers for Disease Control and Prevention
NCHS: National Center for Health Statistics
NHCS: National Hospital Care Survey
NDI: National Death Index
DM: DataMart
sFTP: Secure File Transfer Protocol

## Footnotes

1 HPRNs active in PCORnet from 2016 to 2021.
