# Supplementary material for: Establishing a framework for privacy-preserving record linkage among electronic health record and administrative claims databases within PCORnet®, the National Patient-Centered Clinical Research Network
Source: BMC Res Notes. 2022 Oct 31;15:337. doi: 10.1186/s13104-022-06243-5 (PMC9620597; doi:10.1186/s13104-022-06243-5)
Supplement: Supplementary file 1 — Additional file 1: Figure S1. Overview of the tokenization and linkage flow within PCORnet. The following steps are used to complete the linkage tasks: 1) Run Datavant tokenize. Each site runs Datavant application in tokenize mode on-premises to generate tokens in their own site-specific token encryption scheme. Every site’s tokens are unique. A security breach at one site would not propagate across other sites in the ecosystem. No linking can happen without a site’s permission. 2) Run Datavant transform-tokens. Each site runs Datavant application in transform-tokens to mode to prepare tokens for sending to the Coordinating Center (CC); tokens are uniquely encrypted in transit. 3) Run Datavant transform-tokens. CC runs Datavant application in transform-tokens from to transform tokens into a common CC encryption scheme. Thus, tokens can only be linked at the CC. 4) CC Performs Overlaps. CC runs Datavant Match to determine overlap among records. Figure S2. Overall data flow for the linkage query. Partners generate de-identified tokens from PII held within their source systems (a). The Token Team of the Coordinating Center distributes a SAS query to extract tokens from the HASH_TOKEN table of the CDM (b). Partners execute the query against their CDM and upload the results to a Secure File Transfer location. These tokens are processed by the Datavant software solution (c) and then a Match Index is generated by executing the Datavant Match software (d). A version of this Index is passed to the Query Team of the Coordinating Center that does not include the underlying tokens (e). The Query Team distributes a SAS query to extract Demographic data. Partners execute it against the CDM and the results are returned to a second Secure File Transfer location (f). The results are pulled down by the Query Team and then combined with the token-less Match Index to complete the overlap analysis and to generate the summary demographic table (g). For this study, DCRI acted as the Token [file 13104_2022_6243_MOESM1_ESM.docx]

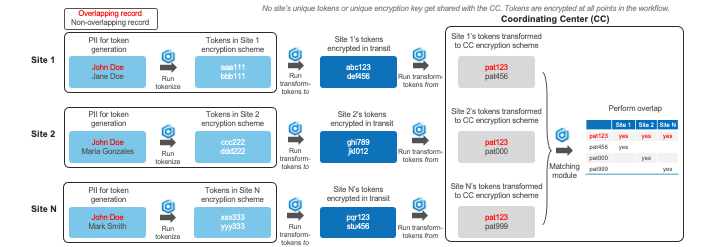


Figure 1:Overview of the tokenization and linkage flow within PCORnet. The following steps are used to complete the linkage tasks: 1) **Run Datavant tokenize.** Each site runs **Datavant** application in tokenize mode on-premises to generate tokens in their own site-specific token encryption scheme. Every site’s tokens are unique. A security breach at one site would not propagate across other sites in the ecosystem. No linking can happen without a site’s permission. 2) **Run Datavant transform-tokens.** Each site runs **Datavant** application in transform-tokens **to** mode to prepare tokens for sending to the Coordinating Center (CC); tokens are uniquely encrypted in transit. 3) **Run Datavant transform-tokens.** CC runs **Datavant** application in transform-tokens **from** to transform tokens into a common CC encryption scheme. Thus, tokens can only be linked at the CC. 4) **CC Performs Overlaps.** CC runs **Datavant** Match to determine overlap among records.

Figure 2: Overall data flow for the linkage query. Partners generate de-identified tokens from PII held within their source systems (a). The Token Team of the Coordinating Center distributes a SAS query to extract tokens from the HASH_TOKEN table of the CDM (b). Partners execute the query against their CDM and upload the results to a Secure File Transfer location. These tokens are processed by the Datavant software solution (c) and then a Match Index is generated by executing the Datavant Match software (d). A version of this Index is passed to the Query Team of the Coordinating Center that does not include the underlying tokens (e). The Query Team distributes a SAS query to extract Demographic data. Partners execute it against the CDM and the results are returned to a second Secure File Transfer location (f). The results are pulled down by the Query Team and then combined with the token-less Match Index to complete the overlap analysis and to generate the summary demographic table (g). For this study, DCRI acted as the Token Team of the Coordinating Center and HPHCI as the Query Team. The number of participating Partners in this initial pilot was 4.

| **DMID_PATID** | **TOKEN_1** | **TOKEN_2** | **TOKEN_N** |
| --- | --- | --- | --- |
| CXYYY_0001 | U3FA3JF39# | 2A93A-A3A93Y | 3Q695U3AFD |
| CXYYY_0002 | AFENAKUT3HO9T | #TJ(G#AGEGAL# | 64GJ(G#AT*Y |
| CXYYY_0003 | %#(#JOYG#O | #$QJ(#PYMhlh | #TMPOYQ#e3qta |

Table 1: Illustration of the content of the HASH_TOKEN extract. There is one row per patient. DMID_PATID corresponds to the ID of the contributing DataMart and PATID corresponds to a pseudoidentifier used to link across all information belonging to a patient within the DataMart’s CDM.

| **MATCH_ID** | **DMID_PATID** | **TOKEN_1** | **TOKEN_2** | **TOKEN_N** |
| --- | --- | --- | --- | --- |
| 0 | C1AAA_0001 | U3FA3JF39# | 2A93A-A3A93Y | 3Q695U3AFD |
| 1 | C1AAA_0002 | AFENAKUT3HO9T |  | 64GJ(G#AT*Y |
| 1 | C2BBB_045 | AFENAKUT3HO9T | #TJ(G#AGEGAL# | 64GJ(G#AT*Y |
| 2 | C3CCC_0100 | J39ANG((#%3Q6Q | AT93G3(2-A0-AT3 | A%)`E@2D@at |
| 2 | C5EEE_4190 |  | AT93G3(2-A0-AT3 | A%)`E@2D@at |

Table 2: Example of matching output. MATCH_ID is used to denote patients that match across DMs. Each MATCH_ID corresponds to a unique patient. DMID_PATID is the internal DM identifier (but does not contain patient identifiers). The TOKEN columns are populated with encrypted hash tokens. MATCH_ID and DMID_PATID are needed to perform the overlap analysis and generate the de-duplicated patient demographic characteristics table.

**Implementation Details**

To support implementation across the network, Datavant provided a number of documents to PCORnet Network Partners. These include security and compliance documentation (e.g., certification of the de-identification software and trusted third party solution, System and Organizational Controls (SOC) 2 compliance, etc.), technical requirements and onboarding instructions. Additional guidance was also developed that described the data inputs required by the software, the token design and composition, details on data governance and enforcement of the token outputs (i.e., inability for sites to generate tokens outside the specified format) and the overall data flow. PCORnet Network Partners implemented version 3.X of the software, though now many have upgraded to v4.x. Partners are able to run Datavant on Mac, Linux or Windows. For SAS, Network Partners may use Windows or Unix/Linux. Partners may be using SAS datasets or views of relational databases. Network Partners must have Base SAS version 9.3 or above. Network Partners were able to their preferred sFTP client to transfer results back to the Coordinating Center.
